# Two Case Reports of Ochrobactrum anthropi Bacteremia in a Tertiary Care Hospital in Northeast India

**DOI:** 10.7759/cureus.59123

**Published:** 2024-04-27

**Authors:** Dipanwita Ray, Stuti Das, Neeta Gogoi, W V Lyngdoh, K G Lynrah

**Affiliations:** 1 Department of Clinical Microbiology, North Eastern Indira Gandhi Regional Institute of Health and Medical Sciences, Shillong, IND; 2 Department of Medicine, North Eastern Indira Gandhi Regional Institute of Health and Medical Sciences, Shillong, IND

**Keywords:** trends of drug resistance, vitek 2 system, emerging pathogen, gram-negative bacteremia, ochrobactrum anthropi

## Abstract

*Ochrobactrum anthropi* is a non-fermenting, Gram-negative bacillus and an emerging opportunistic pathogen. We have isolated this organism from the blood cultures of two patients, a 53-year-old immunocompetent male presenting with an episode of mild fever post craniotomy and an 85-year-old male with chronic obstructive pulmonary disease (COPD) and urinary retention on an indwelling catheter. The organism was identified using VITEK 2 (bioMérieux, France). Both the isolates were resistant to most of the β-lactams, including cephalosporins, and sensitive to quinolones, aminoglycosides, and carbapenems.

## Introduction

The *Ochrobactrum* genus emerged from what was previously identified as Centers for Disease Control and Prevention (CDC) group VD1-2. Initially labeled as *Achromobacter* VD by the Special Bacteriology Section of the US Center for Disease Control, its type species became *O. anthropi* [1]. This aerobic Gram-negative bacterium, non-fermentative in nature, is widely distributed. Its remarkable ability to survive in antiseptic solutions and invasive medical devices, along with its capacity to produce biofilm, contributes to the rising occurrences of hospital-acquired infections [2]. Resistant to various antibiotics, such as beta-lactams, it is increasingly being detected in both immunocompromised and immunocompetent individuals. Consequently, it poses a significant emerging health threat that cannot be ignored any longer [3].

## Case presentation

Case 1

**Figure 1:**
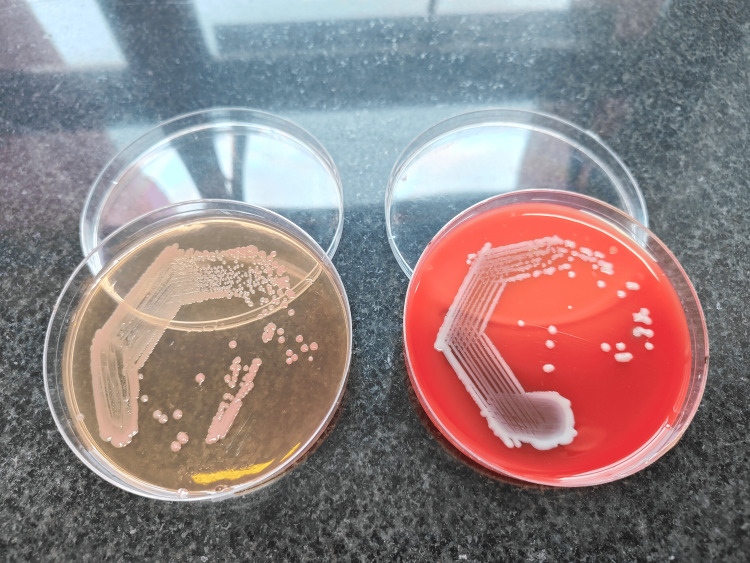
Colonies of Ochrobactrum anthropi on MacConkey and blood agars.

A 53-year-old male was admitted with a left parasagittal extra-axial space-occupying lesion (SOL) and was scheduled for surgery. Following the excision surgery, he developed a mild fever and was subsequently admitted to the intensive care unit (ICU). As shown in Table 1, laboratory investigations revealed hemoglobin levels of 12.3 g%, a total leukocyte count (TLC) of 9900/cu.mm, with a differential leukocyte count (DLC) showing 88% neutrophils (N), 7% lymphocytes (L), 2% eosinophils (E), and 3% monocytes (M), along with a platelet count of 170,000/cu.mm. Paired blood cultures were collected and sent in BacT/ALERT bottle (BioMérieux, France), which flagged positive. Subsequent subcultures on 5% sheep blood agar, chocolate agar, and MacConkey agar plates isolated non-lactose fermenting Gram-negative colonies after 24 hours of incubation at 37°C (Figure 1). The organism displayed motility and tested positive for oxidase and catalase, negative for indole, and positive for urease. The methyl red reaction was negative; it did not utilize citrate and exhibited a typical non-fermentative pattern in triple sugar iron (TSI) tests. These findings raised suspicions, leading to testing in an automated VITEK-2 identification system (BioMérieux, France), which identified it as *O. anthropi* with 98% probability. As susceptibility to *O. anthropi* is not documented in Clinical & Laboratory Standards Institute (CLSI) guidelines, an antibiogram was not provided by VITEK-2. Antimicrobial susceptibility testing (AST) was performed using the Kirby-Bauer disk diffusion method, which revealed sensitivity to ciprofloxacin, levofloxacin, meropenem, imipenem, ertapenem, amikacin, gentamicin, and tetracycline and resistance to ampicillin-sulbactam, amoxicillin-clavulanate, cefotaxime, ceftazidime, cefuroxime, ceftriaxone, cefepime, aztreonam, and piperacillin-tazobactam (Table 2). Accordingly, amikacin therapy was initiated for the patient along with other supportive management, leading to remission of fever and subsequent discharge. A follow-up blood culture collected during a subsequent visit after two weeks showed no bacterial growth after seven days of incubation.

**Table 1:** Laboratory findings of Case 1 and Case 2

Parameters	Case 1	Case 2	Reference range
Hemoglobin	12.3 g/dl	11 g/dl	(15-17) g/dl
Total leukocyte count (TLC)	9900/cu.mm	8100 /cu.mm	(4000-10000) cells/cu.mm
Neutrophils	88%	80%	(40-80)%
Lymphocytes	7%	15%	(20-40)%
Eosinophils	2%	3%	(1-6)%
Monocytes	3%	2%	(2-10)%
Platelet	170,000/cu.mm	200,000/cu.mm	(150,000-400,000) cells/cu.mm

**Table 2:** Antimicrobial susceptibility pattern (N = 2)

Antibiotics	Sensitivity %
Ciprofloxacin	100
Levofloxacin	100
Meropenam	100
Imipenam	100
Ertapenam	100
Amikacin	100
Gentamicin	100
Tetracycline	100
Ampicillin-sulbactam	0
Amoxicillin-clavulanate	0
Cefotaxime	0
Ceftazidime	0
Cefuroxime	0
Ceftriaxone	0
Cefepime	0
Aztreonam	0
Piperacillin-tazobactam	0

Case 2

An 85-year-old man was admitted to the medicine ward presenting with a high fever of 102°F. He had a history of COPD and urinary retention, with intermittent use of a Foley's catheter over the past year. As shown in Table 1, laboratory investigations revealed hemoglobin levels of 11 g%, a TLC of 8100/cu.mm, with a DLC showing 80% neutrophils (N), 15% lymphocytes (L), 3% eosinophils (E), and 2% monocytes (M), along with a platelet count of 160,000/mm^3^. Paired 5% sheep blood cultures sent in BacT/ALERT bottle (BioMérieux) flagged positive, leading to subculture on blood agar, chocolate agar, and MacConkey agar plates. After 24 hours of incubation at 37°C, non-lactose fermenting Gram-negative colonies were isolated. The organism displayed motility and tested positive for oxidase and catalase, negative for indole, and positive for urease. The methyl red reaction was negative; it did not utilize citrate and exhibited a typical non-fermentative pattern in TSI tests. These findings prompted further testing in an automated VITEK-2 identification system (BioMérieux, France), which identified it as OA with 98% probability. AST was performed using the Kirby-Bauer disk diffusion method, indicating sensitivity to ciprofloxacin, levofloxacin, meropenem, imipenem, ertapenem, amikacin, gentamicin, and tetracycline, and resistance to ampicillin-sulbactam, amoxicillin-clavulanate, cefotaxime, ceftazidime, cefuroxime, ceftriaxone, cefepime, aztreonam, and piperacillin-tazobactam (Table 2). Consequently, the patient was initiated on meropenem therapy along with other supportive measures, resulting in the resolution of fever and subsequent discharge. A follow-up blood culture collected during a subsequent visit after three weeks showed no bacterial growth after seven days of incubation.

## Discussion

*Ochrobactrum* spp. is classified within the family Brucellaceae. Within the genus *Ochrobactrum*, there are nine species, but only three, which are *O. anthropi, O. intermedium*, and *O. pseudintermedium,* have been documented to cause infections in humans [4]. Recognized as an emerging opportunistic infection within the last decade, *O. anthropi*, due to its similar environmental niche and biochemical characteristics to *Pseudomonas* (both are oxidase-positive and motile), is often mistaken for the latter, contributing to its underdiagnosis [5]. This study reports two cases encountered within a two-month period in the same year. A hospital infection control program was carried out by environmental sampling in areas where the patients stayed, such as the ICU, medicine ward, and neurosurgery ward, but this yielded no growth of *O. anthropi*. Automation plays a crucial role in accurately identifying such pathogens within a short span of time, which may be overlooked by conventional methods [6].

While most case reports focus on *O. anthropi *infections in immunocompromised hosts, there are instances, such as the case reported by Kettaneh et al., detailing fatal septic shock in an immunocompetent adult caused by *O. anthropi* [7]. In addition, Vaidya et al. reviewed the literature on *O. anthropi* infections in immunocompetent patients [8]. *O. anthropi* typically exhibits robust growth on routine media within 24 hours, forming mucoid, circular colonies approximately 1-2 mm in diameter, which are smooth, shiny, and fully intact (see Figure 1). In our study, both isolates were susceptible to carbapenems, aminoglycosides, and quinolones and responded well to monotherapy. These findings suggest that *O. anthropi *infections may occur frequently in both immunocompromised and immunocompetent individuals. It is crucial to recognize the clinical significance of such unconventional pathogens and utilize modern identification tools for accurate diagnosis. Early identification and appropriate antibiotic therapy are associated with favorable outcomes, as observed in both cases.

## Conclusions

Our findings suggest that *O. anthropi *can cause infections in both immunocompromised and immunocompetent hosts. It is an emerging pathogen that has been found to be resistant to commonly used antibiotics. Hence, it can be a cause of major public health concern in the future. Strict adherence to hospital infection control practices is required. Automated methods for the identification of this emerging pathogen, which is otherwise missed with conventional methods, is the need of the hour.
